# Early neonatal mortality and determinants in sub-Saharan Africa: Findings from recent demographic and health survey data

**DOI:** 10.1371/journal.pone.0304065

**Published:** 2024-06-07

**Authors:** Tadesse Tarik Tamir, Yirgalem Mohammed, Alemneh Tadesse Kassie, Alebachew Ferede Zegeye

**Affiliations:** 1 Department of Pediatric and Child Health Nursing, School of Nursing, College of Medicine and Health Sciences, University of Gondar, Gondar, Ethiopia; 2 Department of Health system and Policy, College of Medicine and Health Science, School of Public Health, Wollo University, Dessie, Ethiopia; 3 Department of Clinical Midwifery, School of Midwifery, College of Medicine and Health Sciences, University of Gondar, Gondar, Ethiopia; 4 Department of medical Nursing, School of Nursing, College of Medicine and Health Sciences, University of Gondar, Gondar, Ethiopia; Arba Minch University, ETHIOPIA

## Abstract

**Introduction:**

Neonatal mortality during the first week of life is a global issue that is responsible for a large portion of deaths among children under the age of five. There are, however, very few reports about the issue in sub-Saharan Africa. For the sake of developing appropriate policies and initiatives that could aid in addressing the issue, it is important to study the prevalence of mortality during the early neonatal period and associated factors. Thus, the aim of this study was to ascertain the prevalence of and pinpoint the contributing factors to early neonatal mortality in sub-Saharan Africa.

**Method:**

Data from recent demographic and health surveys in sub-Saharan African countries was used for this study. The study included 262,763 live births in total. The determinants of early newborn mortality were identified using a multilevel mixed-effects logistic regression model. To determine the strength and significance of the association between outcome and explanatory variables, the adjusted odds ratio (AOR) at a 95% confidence interval (CI) was computed. Independent variables were deemed statistically significant when the p-value was less than the significance level (0.05).

**Result:**

Early neonatal mortality in sub-Saharan Africa was 22.94 deaths per 1,000 live births. It was found to be significantly associated with maternal age over 35 years (AOR = 1.77, 95% CI: 1.34–2.33), low birth weight (AOR = 3.27, 95% CI: 2.16, 4.94), less than four ANC visits (AOR = 1.12, 95% CI: 1.01, 1.33), delivery with caesarean section (AOR = 1.81, 95% CI: 1.30–2.5), not having any complications during pregnancy (AOR = 0.76, 95% CI: 0.61, 94), and community poverty (AOR = 1.32, 95% CI: 1.05–1.65).

**Conclusion:**

This study found that about twenty-three neonates out of one thousand live births died within the first week of life in sub-Saharan Africa. The age of mothers, birth weight, antenatal care service utilization, mode of delivery, multiple pregnancy, complications during pregnancy, and community poverty should be considered while designing policies and strategies targeting early neonatal mortality in sub-Saharan Africa.

## Introduction

The term "early neonatal mortality" (ENM) refers to the death of a newborn that occurs within the first week of life [1]. Although neonatal mortality has decreased significantly over the past few decades, it still poses a serious problem for the majority of low-income countries [2]. The early neonatal period is responsible for around 33% of all under-five deaths globally, whereas the remaining 67% happen after one week of life over their five-year period [3]. Of the 2.8 million babies that die throughout the neonatal period globally each year, 73% do so in the first week after delivery (early neonatal period) [1, 4].

Globally, 2.7 million babies lose their lives within the first month of life due to infections, preterm birth problems, and birth asphyxia. Ninety-nine percent of newborn fatalities and stillbirths take place in low- and middle-income countries like those in sub-Saharan Africa [5]. Sub-Saharan Africa had the highest newborn mortality rate in 2019 with 27 deaths per 1,000 live births, followed by Central and Southern Asia with 24 deaths per 1,000 live births [6]. A child born in sub-Saharan Africa or southern Asia has a tenfold higher probability of dying in the first month of life than a child born in a high-income country [6].

Only one sub-Saharan African country, Madagascar, was found to have met Millennium Development Goal (MDG) 4 in the year 2015 [7]. The Sustainable Development Goals (SDGs) were established during the United Nations meeting in 2015 to succeed the MDGs. Sustainable Development Goal (SDG) three has set a target to reduce mortality in children younger than 5 years to 25 or fewer per 1000 live births and the neonatal mortality rate to 12 or less per 1000 live births by 2030 [8, 9]. Despite the fact that neonatal mortality has decreased significantly since 1990, further efforts are required to accelerate this progress and meet the SDG objective by 2030 [10].

The lack of basic antenatal care [3, 11], complications during pregnancy [12], birth weight [13, 14], cultural practices [15], wealth index [16], home delivery [17], difficulty affording health care [18], and low maternal education status [19] were among the factors contributing to neonatal mortality in low and middle income countries.

The early neonatal period, the first seven days of life, are the most precarious for a baby’s survival [6]. Children face the highest risk of death in their first week of life [6], especially in low- and middle-income countries. To the knowledge of researchers, no study has been conducted on neonatal mortality in the first week of life and determinants at inter-country levels in sub-Saharan Africa. Coming up with its magnitude and determinants helps understand the current status of the problem and can be an input for policymakers. Hence, this study aimed to assess the prevalence and determinants of early neonatal mortality in sub-Saharan Africa.

## Methods

### Data and study setting

The secondary data analysis of recent sub-Saharan Africa (SSA) Demographic Health Survey datasets from 2014 to 2019/20 was conducted (Table 1). The datasets were appended together to investigate early neonatal mortality and determinants in SSA. The data were accessed from the official Demographic Health Survey program database (http://www.dhsprogram.com). The Demographic and Health Surveys (DHS) are surveys that are nationally representative and offer data that is comparable across nations to monitor and assess impact indicators in the areas of population, health, and nutrition. The DHS uses a two-stage stratified cluster sampling technique. The first stage is to select enumeration areas, and the second stage is to draw a sample of households in each enumeration area. We used DHS surveys in 23 sub-Saharan African countries. This study used the children’s record dataset to determine the outcome variable. A sample of 262,763 live births was included in the study (Table 1). While all babies born alive from women of reproductive age five years before the surveys in sub-Saharan Africa were the source population, all live births in the enumeration areas of the survey were the study population.

**Table 1:** Sample size for early neonatal mortality in sub-Saharan Africa countries (n = 262,763).

Country	Year of survey	Frequency	Percent
Angola	2015	14,322	5.45
Benin	2017/18	13,589	5.17
Burundi	2016/17	13,192	5.02
Cameron	2018	9,733	3.7
Chad	2014/15	18,623	7.09
Ethiopia	2016	10,641	4.05
Gambia	2019	8,362	3.18
Ghana	2015	5,884	2.24
Guinea	2018	7,951	3.03
Kenya	2014	20,964	7.98
Lesotho	2014	3,138	1.19
Liberia	2019/20	5,704	2.17
Malawi	2015	17,286	6.58
Mali	2018	9,940	3.78
Nigeria	2018	33,924	12.91
Rwanda	2019/20	8,092	3.08
Senegal	2019	6,125	2.33
Serra Leone	2019	9,899	3.77
South Africa	2016	3,548	1.35
Tanzania	2015	10,233	3.89
Uganda	2016	15,522	5.91
Zambia	2018	9,959	3.79
Zimbabwe	2015	6,132	2.33
Total sample size	2014-2019/20	262,763	100

### Measures

The outcome variable for this study was early neonatal mortality, which was dichotomized as "yes" = 1 for neonates who died within one week of life and "no" = 0 for neonates who were alive within one week of life. Considering the hierarchical DHS data, two-level explanatory variables (individual and community) were used to identify the determinants of early neonatal mortality. The individual-level variables included maternal age, maternal education, place of delivery, birth weight, type of pregnancy, mode of delivery, household wealth index, birth order, type of pregnancy, antenatal care (ANC) utilization, and pregnancy complications. In this study, missing values have been managed by dropping variables assigned as don’t know and unspecified numbers.

Place of residence, country, community illiteracy level, and community poverty level make up the community-level variables. The individual-level variables of maternal education and household wealth index, respectively, were aggregated to determine the community levels of illiteracy and poverty.

### Statistical analysis

Once extracted from the source, the data from each country has been appended together to create one single file dataset using Stata 14. To draw valid inferences, the weighting was done using the weighting variables [sample weight (v005), primary sampling unit (v023), and stratum (v021)]. Because DHS data is hierarchical in nature, a multilevel mixed effect logistic regression model was applied to assess determinants of early neonatal mortality in SSA.

Multilevel mixed effect logistic regression has four models; the null model, model I, model II, and model III. Null model is used to determine the applicability of multilevel to the data by determining measure of variation. Model I uses to determine the effect of only individual level factors on early neonatal mortality. Model II uses to determine the effect of community level factors on early neonatal mortality. The last model (model III) shows the effects of both individual and community level factors on the mortality. Multilevel logistic regression is equated as follows,

$$\log\left(\frac{\Pi ij}{1-\Pi ij}\right)=\beta o+\beta 1x1ij+\cdots +\beta nxnij+uoj+eij$$

Where; πij is the probability of baby to die within one week of life, (1-πij) is the probability of baby not to die within one week of life, βo is log odds of the intercept, β1, … βn are effect sizes of individual and community-level factors, x1ij … xnij are independent variables of individuals and communities. The quantities uoj and eij are random errors at cluster levels and individual levels respectively.

The measurement of variation (random effect) was done using the median odds ratio (MOR), intra-cluster correlation coefficient (ICC), and proportional change in variation (PCV). The MOR is the unexplained heterogeneity of early neonatal mortality between clusters, and it shows the odds of variation in early neonatal mortality between high- and low-risk clusters, taking two clusters at random. ICC indicates the percent of variation in early neonatal mortality between clusters, and PCV is the percent of variation in early neonatal mortality attributed to individual and community-level factors. The parameters MOR, ICC, and PCV were determined by the following equations: MOR = exp.(0.95√VC), $ICC=\frac{VC}{vC}+3.29\times 100\text{\%}$ and $PCV=\frac{VNull-VC}{VNull}\times 100\text{\%}$, where; VC = variance of the cluster for respective model and Null = variance of the null model.

The measure of association or fixed effect was determined by the computation of the AOR at 95% CI and p-value. The best-fit model was selected by using deviance and log likelihood ratio (LLR). A model with a small value of deviance (-2×LLR) and a large value of log likelihood ratio was taken as the best fit.

### Ethical consideration

The data for this study were extracted from the recent DHS dataset. The only prerequisite for access to the DHS data is registration. Therefore, receiving ethical approval for this study was not appropriate. More details regarding DHS data and ethical standards are available online at (http://www.dhsprogram.org.com).

## Results

### Socio-demographic characteristics of the study subjects

A total of 262,763 weighted live births were enrolled in the analysis of this study to determine the death of neonates within the first week of life in sub-Saharan Africa. The mean (±SD) age of mothers of babies who were study subjects was 29±7 years. Nearly three-fourths (75.29%) of babies were born to mothers in the age group of 20–35 years. One hundred twenty-four thousand and seven hundred sixty-two (47.48%) of the study subjects were from households of poor wealth status. About 70% of study subjects resided in rural areas of sub-Saharan Africa. Out of the total live births included in the study, 112,021 (42.63%) were from the eastern part of Africa (Table 2).

**Table 2:** Socio-demographic characteristics of the study subjects.

Variables	Category	Frequency(n)	Percent (%)
**Individual level variables**			
Maternal age	15–19	15,598	5.94
	20–35	197,846	75.29
	36–49	49,319	18.77
Maternal education	Unable to read and write	102,849	39.14
	Primary	89,884	34.21
	Secondary and above	70,028	26.65
Birth weight	Low	13,225	9.68
	Normal	103,531	75.79
	High	19,843	14.53
Household wealth index	Poor	124,762	47.48
	Middle	52,216	19.87
	Rich	85,785	32.65
ANC visits	<4 visits	77,104	43.26
	≥4 visits	101,134	56.74
Birth order	First	57,299	21.81
	Second	50,346	19.16
	Third	42,245	16.08
	Fourth or more	112,873	42.96
Mode of delivery	Cesarean section	13,085	4.99
	Vaginal	249,125	95.01
Pregnancy complications	Yes	16,805	58.72
	No	11,815	41.28
Place of delivery	Home	94,244	36.35
	Health facility	165,046	63.65
Multiple pregnancy	Single	253,579	96.5
	Multiple	9,184	3.5
**Community level variables**			
Residence	Urban	79,400	30.22
	Rural	183,363	69.78
Country category	Central Africa	42,678	16.24
	East Africa	112,021	42.63
	West Africa	101,378	38.58
	Southern Africa	6,686	2.54
Community level illiteracy	Low	114,071	43.41
	High	148,692	56.59
Community level poverty	Low	128,978	49.09
	High	133,785	50.91

### Prevalence of early neonatal mortality in sub-Saharan Africa

**Fig 1:**
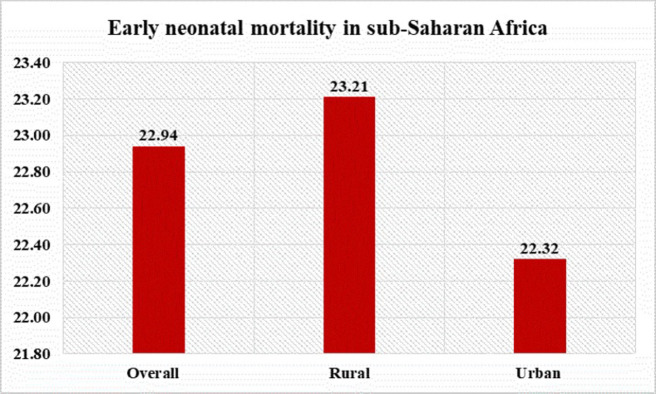
Prevalence of early neonatal mortality in sub-Saharan Africa.

Prevalence of early neonatal mortality in sub-Saharan Africa was found to be 22.94 deaths per 1000 live births at 95% CI (22.38, 23.5). Early neonatal mortality in urban and rural areas of sub-Saharan Africa was 22.32 and 23.21 deaths per 1000 live births (Fig 1).

### Measure of variation and model fit statistics

As shown in Table 3 below, 10% of the variation in early neonatal mortality happened between clusters, and the rest of the variation occurred within clusters (ICC = 10%). The MOR in the null model shows that the odds of early neonatal mortality were 1.77 times higher among clusters of high risk for mortality compared to clusters of low risk for mortality. In model I, about 43% of the variation in early neonatal mortality was attributed to individual-level factors (PCV = 42.5%). In the final model, 67% of the variation in early neonatal mortality was due to both individual and community-level factors included in the regression. The odds of early neonatal mortality were 1.39 times higher among high-risk clusters compared to low-risk clusters (MOR = 1.39 in model III). According to the model fit statistics of this study, model III had the lowest deviance and a large LLR value, which indicated that it was the best fitted model.

**Table 3:** Measure of variation and model fit statistics of early neonatal mortality in sub-Saharan Africa.

Parameter	Null model	Model I	Model II.	Model III
**Measure of variation**				
**Variance**	0.362162	0.2079343	0.254229	0.1199074
**ICC**	10.0%	6.0%	7%	4%
**MOR**	1.77	1.54	1.61	1.39
**PCV**	Reference	42.5%	29.8%	67.1%
**Model fitness**				
**LLR**	-28701.5	-1892.4	-28629.9	-1877.4
**Deviance**	57403.0	3784.8	57259.8‬	3754.8

ICC: intra-cluster correlation coefficient, MO: median odds ratio, PCV: proportional change in variance and LLR: log likelihood ratio.

### Measure of association of early neonatal mortality in sub-Saharan Africa

A total of 14 variables (individual and community) were included in final model (Model III) to identify determinants of early neonatal mortality in SSA. Namely; maternal age, maternal education, birth weight, household wealth index, ANC visits, pregnancy complications, place of delivery, type of pregnancy, birth order, mode of delivery, place of residence, country category, community level illiteracy, community level poverty (Table 4).

**Table 4:** Measure of association of early neonatal mortality in sub-Saharan Africa.

Individual and community level factors		Model I AOR(95% CI)	Model II AOR(95% CI)	Model III AOR(95% CI)
Maternal age	15–19	1.12 (0.88, 1.44)		1.30(0.20, 1.94)
	20–35	1		1
	36–49	1.53 (1.30, 1.80)		1.77(1.34, 2.33)*
Maternal education	No formal education	0.80 (0.67, 0.95)		0.83 (0.69, 1.01)
	Primary	0.88 (0.74, 1.04)		0.92 (0.77, 1.09)
	Secondry and above	1		1
Birth weight	Low	3.13 (2.53, 3.90)		3.27 (2.16, 4.94)*
	Normal	1		1
	High	2.37 (0.85, 3.02)		2.96 (0.94, 4.51)
Household wealth index	Poor	0.91 (0.78, 1.05))		1.12 (0.84, 1.51)
	Middle	1.05 (0.88, 1.25)		1.16 (0.85, 1.6)
	Rich	1		1
ANC visits	<4 visits	1.15 (1.01, 1.31))		1.14 (0.91, 1.42)*
	≥4 visits	1		1
Pregnancy complications	Yes	1		1
	No	1.01 (0.90, 1.15)		0.76 (0.61, 0.94)*
Place of delivery	Home	3.8 (0.83, 4.02)		3.59 (0.73, 4.73)
	Facility	1		1
Multiple Pregnancy	Multiple	4.65 (3.74, 5.83)		4.02 (2.66, 6.08)*
	Single	1		1
Birth order	First	1.29 (0.92, 1.81)		1.25 (0.89, 1.769)
	Second	1.01 (0.72, 1.42)		0.99 (0.70, 1.39)
	Third	1.01 (0.71, 1.41)		0.99 (0.71, 1.39)
	Fourth or more	1		1
Mode of delivery	Cesarean section	2.05 (1.68, 2.51)		1.81 (1.30, 2.50)*
	Vaginal	1		1
Place of residence	Urban		1	1
	Rural		1.05 (1.01, 1.11)	1.11 (0.85, 1.46)
Part of sub-Saharan Africa	Central Africa		1.08 (1.01, 1.15)	1.17 (0.88, 1.57)
	East Africa		1	1
	West Africa		1.10 (0.96, 1.26)	1.09 (0.81, 1.45)
	Southern Africa		1.24 (0.7, 1.41)	2.60 (0.8, 3.76)
Community level illitracy	Low		1	1
	High		1.04 (0.98, 1.11)	0.90(0.71, 1.13)
Community level poverity	Low		1	1
	High		1.01 (0.94, 1.065)	1.32 (1.05, 1.65)*

ANC: antenatal care, CS: caesarean section

*: level of significance (p value) less than 0.05.

The output result of the final model (Model III) of multilevel mixed effect logistic regression shows that maternal age, birth weight, number of ANC visits, mode of delivery, pregnancy complications, multiple pregnancy and community level poverty were significantly associated with early neonatal mortality (Table 4).

The odds of early neonatal mortality were 1.77 times higher for babies born to mothers in the age group of 36–49 (AOR = 1.77, 95% CI: 1.34–2.33) compared to babies born to mothers in the age group of 25–35. For low-birth-weight babies, the odds of early neonatal mortality were 3.27 times higher (AOR = 3.27, 95% CI: 2.16, 4.94) compared to those born with normal birth weight. The odds of death within the first week of life were 1.12 times higher for babies born to mothers who had less than four ANC visits during pregnancy (AOR = 1.12, 95% CI: 1.01, 1.33), taking babies born to mothers who had four and above ANC visits as a reference.

For babies delivered by caesarean section, the odds of early neonatal mortality were 1.81 (AOR = 1.81; 95% CI: 1.30–2.5) times higher compared to babies delivered vaginally. In reference to babies born to mothers with complicated pregnancies, the odds of early neonatal mortality among babies born with no complications during pregnancy were reduced by 24% (AOR = 0.76, 95% CI: 0.61, 0.94). The odds of early neonatal mortality were 4.02 times higher (AOR = 4.02, 95% CI: 2.66, 6.08) for neonates born multiple compared to those born singleton. Taking babies born in communities with low levels of poverty as reference, the odds of early neonatal mortality were 1.32 (AOR = 1.32, 95% CI: 1.05–1.65) times higher among babies born in communities with high levels of poverty (Table 4).

## Discussion

The death of neonates in the early stages of their lives is a global public health concern, especially in developing countries like sub-Saharan Africa. Using data from the recent demographic and health survey, this study determined the prevalence and associated factors of early neonatal mortality in sub-Saharan Africa.

The prevalence of early neonatal mortality in this study was found to be 22.94 deaths per 1000 live births at a 95% CI (22.38, 23.5). This implies that the rate of early neonatal mortality in sub-Saharan Africa was about twofold higher than the Sustainable Development Goal (SDG) of 2030 to reduce the rate of neonatal mortality to 12 or less per 1000 live births [8].

On the one hand, the prevalence of early neonatal mortality in our study was lower than the prevalence in Nigeria, with 32 deaths per 1000 live births. This lower prevalence of early neonatal mortality than in the Nigerian study could be related to the DHS data used. Our study was conducted using DHS data from 2014 to 2020, whereas the study in Nigeria used DHS 2013. In this developing world, the rate of mortality among neonates decreases with time due to the advancement of technology and quality maternal and child health services. The more recent the DHS data is, the lower the rate of mortality could be.

On the other hand, the prevalence in this study was higher than the study in Afghanistan with prevalence of 14 deaths per 1000 live births [2]. This discrepancy between prevalence in our study and Afghanistan could be due to the variation in health infrastructure and socio-economic status across countries. In addition, according to 2018 United Nations estimates, Afghanistan has witnessed about a 50% and 62% reduction in maternal and child mortality from 1990 to 2017, respectively.

The multivariable multilevel mixed effect logistic regression analysis of this study revealed that maternal age, birth weight, ANC visit, multiple pregnancies, mode of delivery, pregnancy complications, and community level of poverty were significantly associated with early neonatal mortality.

The odds of early neonatal mortality were higher for babies born to mothers aged over 35 years compared to babies born to mothers in age group of 25–35 years. Previous studies have also reported the same [2, 20]. The significant association between early neonatal mortality and maternal age above 35 could be due to the fact that low-birth-weight and fetal macrosomia is more prevalent among baies born to mothers of advanced ages [20, 21].

Regarding birth weight, the odds of early neonatal death were higher among low-birth-weight babies compared to babies of normal birth weight. This is consistent with previous studies [21, 22]. One of the common reasons for higher odds of early neonatal mortality among low birth weight babies is that most of the time, low birth weight babies are preterm births and/or small for gestational age [21]. These findings suggest the need to improve mother care during pregnancy, childbirth, and postnatal periods, particularly for low birth weight babies. The World Health Organisation has also developed clinical guidelines to increase baby survival and advocates the need for carefull essential newborn care for low-birth-weight babies [23]. Kangaroo mother care can be an option for neonatal care with low birth weights; it involves skin-to-skin contact between a mother and her newborn [24].

The antenatal care visits during pregnancy of this birth was significantly associated with early neonatal mortality. The odds of death within the first week of life were higher for babies born to mothers who had less than four ANC visits during pregnancy, taking babies born to mothers who had four and above ANC visits as a reference group. It was evidenced that ANC service utilization only reduces mortality of neonates by an estimate of 10–20% [25, 26], although there is insufficient utilization of ANC services in sub-Saharan Africa [27]. A study conducted to determine the impact of ANC on neonatal mortality also found that "the utilisation of at least one antenatal care visit by a skilled provider during pregnancy reduces the risk of neonatal mortality by 39% in sub-Saharan African countries’ [28].

Surprisingly, this study found that cesarean section as a mode of delivery was significantly associated with early neonatal mortality. Though a cesarean section is performed to save the life of the newborn, the odds of early neonatal mortality were higher among babies delivered by cesarean section compared to babies delivered vaginally. This finding was coherent with the previous studies [29–31]. On this basis, previous scientific literature also recommends avoiding cesarean sections as intrapartum interventions when there is no clear medical indication that they will improve the outcome for the mother or the baby [30, 32, 33]. However, it could be plausible that the higher odds of early neonatal mortality among babies born via cesarean section were due to the fact that the majority of CS deliveries occur as a last option for delivery when there are pregnancy complications. The association between pregnancy complications and early neonatal mortality in our study supports this justification.

In this study, pregnancy comlication was another factor significantly associated with early neonatal mortality. In reference to babies born to mothers with complicated pregnancies, the odds of early neonatal mortality among babies born with no complications during pregnancy were reduced by 24%. This finding was supported by previous studies [34, 35]. Hence, prevention and carefully tackling complications that happen during pregnancy could be essential strategies to reduce mortality in newborns.

Furthermore, multiple pregnancy was significanly associated with early neonatal mortality. The odds of early neonatal mortality was four times higher for neonates born multiple compared to those born singleton. This finding agrees with previous study [36]. It could be logical explanation that babies born from multiple pregnancies typically suffer growth constraints, low Apgar scores, and very low birth weights [37, 38]. Moreover, multiple pregnancies are more likely to be complicated during pregnancy, labor and after delivery [38].

Eventually, community poverty level was significantly associated with early neonatal mortality. Accordingly, the odds of early neonatal mortality increased by 32 percent among babies born in communities with high levels of poverty, taking babies born in communities with low levels of poverty as a reference group. This finding was supported by previous evidence [39–41]. Babies who die in early neonatal life suffer from conditions and diseases associated with a lack of quality care at or immediately after birth and in the first week of life [42]. Poverty can exacerbate these issues by limiting access to quality healthcare, nutrition, and sanitation, which can increase the risk of neonatal mortality [42].

The use of nationally collected large samples of recent demographic and health survey data from sub-Saharan African countries and the application of an appropriate advanced multilevel mixed effect model were the strengths of this study. However, a few sub-Saharan African countries that had not had a demographic or health survey since 2014 were not included, which may have affected the generalizability of our finding. In addition, the study used DHS data collected in different years, which might have exposed the findings of the study to the differential time effect.

## Conclusion

This study found that about twenty-three neonates out of one thousand live births died within the first week of life in sub-Saharan Africa. The age of mothers, birth weight, antenatal care service utilization, mode of delivery, multiple pregnancy, complications during pregnancy, and community poverty should be considered while designing policies and strategies targeting early neonatal mortality in sub-Saharan Africa.

## Abbreviations

ANC: Antenatal care
AOR: Adjusted odds ratio
CI: Confidence Interval
CS: Caesarean Section
DHS: Demographic and Health Survey
ICC: Intracluster Correlation Coefficient
MOR: Median Odds Ratio
LLR: Log likelihood Ratio
PCV: Proportional Change in Variance
SDG: Sustainable Development Goal

## References

[1] Lehtonen L., et al. Early neonatal death: a challenge worldwide. in Seminars in Fetal and Neonatal Medicine. 2017. Elsevier. doi: 10.1016/j.siny.2017.02.006 28238633

[2] Kibria G.M.A., et al., Determinants of early neonatal mortality in Afghanistan: an analysis of the Demographic and Health Survey 2015. Globalization and health, 2018. 14(1): p. 1–12. 29743085 10.1186/s12992-018-0363-8 PMC5944060

[3] Dahiru T., Determinants of early neonatal mortality in Nigeria: results from 2013 Nigeria DHS. Journal of Pediatrics & Neonatal Care, 2015. 2(5).

[4] Lawn J.E., et al., Every Newborn: progress, priorities, and potential beyond survival. The lancet, 2014. 384(9938): p. 189–205. doi: 10.1016/S0140-6736(14)60496-7 24853593

[5] Arsenault C., et al., Equity in antenatal care quality: an analysis of 91 national household surveys. The Lancet Global Health, 2018. 6(11): p. e1186–e1195. doi: 10.1016/S2214-109X(18)30389-9 30322649 PMC6187112

[6] Newborns, W., Improving survival and well-being. World Health Organization. https://www.who.int/news-room/fact-sheets/detail/newborns-reducing-mortality, 2020.

[7] Moyer C.A., Dako-Gyeke P., and Adanu R.M., Facility-based delivery and maternal and early neonatal mortality in sub-Saharan Africa: a regional review of the literature. African journal of reproductive health, 2013. 17(3): p. 30–43. 24069765

[8] Transforming our world Cf, O.,: the 2030 Agenda for Sustainable Development. United Nations: New York, NY, USA, 2015.

[9] Sudfeld C.R. and Fawzi W.W., Importance of innovations in neonatal and adolescent health in reaching the sustainable development goals by 2030. JAMA pediatrics, 2017. 171(6): p. 521–522. doi: 10.1001/jamapediatrics.2017.0261 28384685

[10] Hug L., et al., National, regional, and global levels and trends in neonatal mortality between 1990 and 2017, with scenario-based projections to 2030: a systematic analysis. The Lancet Global Health, 2019. 7(6): p. e710–e720. doi: 10.1016/S2214-109X(19)30163-9 31097275 PMC6527519

[11] Amouzou A., et al., Skilled attendant at birth and newborn survival in Sub–Saharan Africa. Journal of global health, 2017. 7(2). doi: 10.7189/jogh.07.020504 29423181 PMC5804504

[12] Titaley C.R., et al., Determinants of neonatal mortality in Indonesia. BMC public health, 2008. 8(1): p. 1–15. doi: 10.1186/1471-2458-8-232 18613953 PMC2478684

[13] Reyes J., et al., Neonatal mortality and associated factors in newborn infants admitted to a Neonatal Care Unit. Arch Argent Pediatr, 2018. 116(1): p. 42–48. 29333811 10.5546/aap.2018.eng.42

[14] Smeeton N.C., et al., Assessing the determinants of stillbirths and early neonatal deaths using routinely collected data in an inner city area. BMC medicine, 2004. 2(1): p. 1–7. 15238165 10.1186/1741-7015-2-27 PMC471578

[15] Wolde H.F., et al., Factors affecting neonatal mortality in the general population: evidence from the 2016 Ethiopian Demographic and Health Survey (EDHS)—multilevel analysis. BMC research notes, 2019. 12(1): p. 1–6. 31547855 10.1186/s13104-019-4668-3 PMC6757386

[16] Organization, W.H., Reaching the every newborn national 2020 milestones: country progress, plans and moving forward. 2017.

[17] Akinyemi J.O., Bamgboye E.A., and Ayeni O., Trends in neonatal mortality in Nigeria and effects of bio-demographic and maternal characteristics. BMC pediatrics, 2015. 15(1): p. 1–12. 25886566 10.1186/s12887-015-0349-0 PMC4395970

[18] Rarani M.A., et al., Changes in socio-economic inequality in neonatal mortality in Iran between 1995–2000 and 2005–2010: an Oaxaca decomposition analysis. International journal of health policy and management, 2017. 6(4): p. 219. doi: 10.15171/ijhpm.2016.127 28812805 PMC5384984

[19] Yaya Y., et al., Maternal and neonatal mortality in south-west Ethiopia: estimates and socio-economic inequality. PloS one, 2014. 9(4): p. e96294. doi: 10.1371/journal.pone.0096294 24787694 PMC4005746

[20] Kim Y.-N., et al., Maternal age and risk of early neonatal mortality: a national cohort study. Scientific reports, 2021. 11(1): p. 1–9. 33436971 10.1038/s41598-021-80968-4 PMC7804272

[21] Suparmi S., Chiera B., and Pradono J., Low birth weights and risk of neonatal mortality in Indonesia. Health Science Journal of Indonesia, 2016. 7(2): p. 113–117.

[22] Migoto M.T., et al., Early neonatal mortality and risk factors: a case-control study in Paraná State. Revista Brasileira de Enfermagem, 2018. 71: p. 2527–2534. 30304186 10.1590/0034-7167-2016-0586

[23] Soon B.T., The global action report on preterm birth. Geneva: World Health Organization, 2012: p. 2.

[24] Conde-AgudeloA D.-R.J. and Belizan J., Kangaroo mother care to reduce morbidity and mortality in low birthweight infants (Review). The Cochrane Collaboration. 2005, Wiley. 10.1002/14651858.CD002771 11034759

[25] Organization, W.H., Women and health: today’s evidence tomorrow’s agenda. 2009: World Health Organization.

[26] Darmstadt G.L., et al., Evidence-based, cost-effective interventions: how many newborn babies can we save? The Lancet, 2005. 365(9463): p. 977–988. doi: 10.1016/S0140-6736(05)71088-6 15767001

[27] UNICEF, Global databases, 2016, based on Multiple Indicator Cluster Surveys (MICS). Demographic and Health Surveys (DHS) and other nationally representative sources, 2016.

[28] Tekelab T., et al., The impact of antenatal care on neonatal mortality in sub-Saharan Africa: A systematic review and meta-analysis. PloS one, 2019. 14(9): p. e0222566. doi: 10.1371/journal.pone.0222566 31518365 PMC6743758

[29] Briand V., et al., Maternal and perinatal outcomes by mode of delivery in senegal and mali: a cross-sectional epidemiological survey. 2012. 10.1371/journal.pone.0047352 PMC3466276 23056633

[30] Gregory K.D., et al., Cesarean versus vaginal delivery: whose risks? Whose benefits? American journal of perinatology, 2012. 29(01): p. 07–18. doi: 10.1055/s-0031-1285829 21833896

[31] Althabe F., et al., Cesarean section rates and maternal and neonatal mortality in low‐, medium‐, and high‐income countries: an ecological study. Birth, 2006. 33(4): p. 270–277. doi: 10.1111/j.1523-536X.2006.00118.x 17150064

[32] Deneux-Tharaux C., et al., Postpartum maternal mortality and cesarean delivery. Obstetrics & Gynecology, 2006. 108(3 Part 1): p. 541–548. doi: 10.1097/01.AOG.0000233154.62729.24 16946213

[33] Villar J., et al., Maternal and neonatal individual risks and benefits associated with caesarean delivery: multicentre prospective study. Bmj, 2007. 335(7628): p. 1025. doi: 10.1136/bmj.39363.706956.55 17977819 PMC2078636

[34] Schoeps D., et al., Risk factors for early neonatal mortality. Revista de saude publica, 2007. 41: p. 1013–1022. 18066471 10.1590/s0034-89102007000600017

[35] Nakimuli A., et al., Still births, neonatal deaths and neonatal near miss cases attributable to severe obstetric complications: a prospective cohort study in two referral hospitals in Uganda. BMC pediatrics, 2015. 15: p. 1–8. 25928880 10.1186/s12887-015-0362-3 PMC4416266

[36] Bellizzi S., et al., Early neonatal mortality in twin pregnancy: findings from 60 low-and middle-income countries. Journal of global health, 2018. 8(1). doi: 10.7189/jogh.08.010404 29423189 PMC5782831

[37] Vogel J.P., et al., Maternal and perinatal outcomes of twin pregnancy in 23 low-and middle-income countries. PloS one, 2013. 8(8): p. e70549. doi: 10.1371/journal.pone.0070549 23936446 PMC3731264

[38] Nwankwo T., et al., Pregnancy outcome and factors affecting vaginal delivery of twins at University of Nigeria Teaching Hospital, Enugu. Nigerian journal of clinical practice, 2013. 16(4). doi: 10.4103/1119-3077.116895 23974745

[39] Kenner C., Sugrue N.M., and Finkelman A., Poverty and neonatal outcomes: how nurses around the world can make a difference. Nursing for Women’s Health, 2007. 11(5): p. 468–473. doi: 10.1111/j.1751-486X.2007.00214.x 17897426

[40] Mohamoud Y.A., Kirby R.S., and Ehrenthal D.B., Poverty, urban-rural classification and term infant mortality: a population-based multilevel analysis. BMC pregnancy and childbirth, 2019. 19: p. 1–11. 30669972 10.1186/s12884-019-2190-1 PMC6343321

[41] Thomson K., et al., Socioeconomic inequalities and adverse pregnancy outcomes in the UK and Republic of Ireland: a systematic review and meta-analysis. BMJ open, 2021. 11(3): p. e042753. doi: 10.1136/bmjopen-2020-042753 33722867 PMC7959237

[42] Watkins K., The State of the World’s Children 2016: A Fair Chance for Every Child. 2016: ERIC.

