# Supplementary material for: Early neonatal mortality and determinants in sub-Saharan Africa: Findings from recent demographic and health survey data
Source: PLoS One. 2024 Jun 7;19(6):e0304065. doi: 10.1371/journal.pone.0304065 (PMC11161111; doi:10.1371/journal.pone.0304065)
Supplement: Attachment — Submitted filename: PLOS ONE REGISTERED REPORT PROTOCOL REVIEWER.docx [file pone.0304065.s001.docx]

**Reviewer Recommendation and Comments for Manuscript Number**

PONE-D-23-16077

Does the manuscript provide a valid rationale for the proposed study, with clearly identified and justified research questions?

The research question outlined is expected to address a valid academic problem or topic and contribute to the base of knowledge in the field.

The research provides a valid rationale for the proposed study with a clearly identified research though

2. Is the protocol technically sound and planned in a manner that will lead to a meaningful outcome and allow testing the stated hypotheses?

The manuscript should describe the methods in sufficient detail to prevent undisclosed flexibility in the experimental procedure or analysis pipeline, including sufficient outcome-neutral conditions (e.g. necessary controls, absence of floor or ceiling effects) to test the proposed hypotheses and a statistical power analysis where applicable. As there may be aspects of the methodology and analysis which can only be refined once the work is undertaken, authors should outline potential assumptions and explicitly describe what aspects of the proposed analyses, if any, are exploratory.

The work is technically sound though the following need to be addressed;

**Methods**

- Use of Kids Records is not the most appropriate word to use unless it was officially called that as in statement below;

This study used the Kids Records (KR) dataset to determine the outcome variable.

The two statements are contradictory, address the discrepancy;

- A sample of 262,763 live births were included in the study (Table 1).

Table 1: Sample size for early neonatal mortality in Sub-Saharan Africa countries (n=352,605).

- Be consistent use either subject or participants.

3. Is the methodology feasible and described in sufficient detail to allow the work to be replicable?

There is need to provide more detailed information about the inclusion criteria

- Describe the inclusion criteria in this study and how you deal with missing data which a common problem with secondary data

4. Have the authors described where all data underlying the findings will be made available when the study is complete?

- The PLOS Data policy requires authors to make all data underlying the findings described in their manuscript fully available without restriction, with rare exception, at the time of publication. The data should be provided as part of the manuscript or its supporting information, or deposited to a public repository. For example, in addition to summary statistics, the data points behind means, medians and variance measures should be available. If there are restrictions on publicly sharing data—e.g. participant privacy or use of data from a third party—those must be specified.
- Participants have not described how data will be made available

5. Is the manuscript presented in an intelligible fashion and written in standard English?

*PLOS ONE* does not copyedit accepted manuscripts, so the language in submitted articles must be clear, correct, and unambiguous. Any typographical or grammatical errors should be corrected at revision, so please note any specific errors here.

Discussion

- Paragraph your work well
- Work on spacing between the reference and the words before
- Ensure that the font size is the same throught the document see the paragraph below

In this study, pregnancy complication was another factor significantly associated with early neonatal mortality. In reference to babies born to mothers with complicated pregnancies, the odds of early neonatal mortality among babies born with no complications during pregnancy were reduced by 24%. This finding was supported by previous studies (29, 30)

6. Review Comments to the Author

Please use the space provided to explain your answers to the questions above and, if applicable, provide comments about issues authors must address before this protocol can be accepted for publication. You may also include additional comments for the author, including concerns about research or publication ethics.You may also provide optional suggestions and comments to authors that they might find helpful in planning their study.

(Please upload your review as an attachment if it exceeds 20,000 characters) (Limit 100 to 20000 Characters)

Some comments have been placed in the different sections

**Introduction**

- Number the lines so that they are easy to refer to
- Be specific for example when you stated in the introduction that;
- Before turning five, 3.3 million more children will die(4). Specify what measurement five is … days, months..years etc
- Please talk of SDG because these are the ones currently being referred to ..
- There many grammatically errors
- Provide references for the paragraph below;
- The early neonatal period, the first seven days of life, are the most precarious for a baby's survival. Children face the highest risk of death in their first week of life, especially in low- and middle income countries.
- Provide references to qualify the paragraph below;

Since neonatal mortality in the first week of life is understudied in sub-Saharan

Africa, coming up with its magnitude and determinants helps understand the current status of

**Results**

- Clarify on what participants ( mothers or neonates) with mean/SD of 29±7 standard deviation (SD). you were referring to in the statement below

neonates within the first week of life in sub-Saharan Africa. The mean age of participants was

29±7 standard deviation (SD). Nearly three-fourths (75.29%) of babies in this study were born to mothers in the age group of 20–35 years. One hundred twenty-four thousand and seven hundred

- State the units of measurement used in The mean age of participants was 29±7 standard deviation (SD).
- Figure 1 is missing
- Bold the titles of the tables so that they can easily be identified….
- Leave space between the narratives before the tables and the tables
- Spelling of maternal education in table 4
- Table 4 should reflect the outcome as in two by two format. Ie outcome vs independent variables under the different models

*7. PLOS authors have the option to publish the peer review history of their article (what does this mean?). If published, this will include

your full peer review and any attached files.

If you choose “no”, your identity will remain anonymous but your review may still be made public.

**Do you want your identity to be public for this peer review?** For information about this choice, including consent withdrawal, please

- No

***Confidential to Editor**

1. Do you have any potential or perceived competing interests that may influence your review? Please review our Competing Interests policy and declare any potential interests that you feel the Editor should be aware of when considering your review. If you have no competing interests, please write "I have no competing interests." (Limit 20000 Characters)

- I don’t have any competing interests

2. Did you receive any assistance in preparing this review (e.g. from a post-doc or graduate student)? If yes, please provide his/her name and title here. If you have chosen to reveal your identity to the authors in question 6 above, please include the name of the person who assisted you in the response to question 6 as well. (optional) (Limit 200 Characters)

- I didnt receive any assistance in preparing this review
