# Supplementary material for: Early neonatal mortality and determinants in sub-Saharan Africa: Findings from recent demographic and health survey data
Source: PLoS One. 2024 Jun 7;19(6):e0304065. doi: 10.1371/journal.pone.0304065 (PMC11161111; doi:10.1371/journal.pone.0304065)
Supplement: Attachment — Submitted filename: Rebutal letter.docx [file pone.0304065.s003.docx]

**Response to comments**

**Manuscript ID:** PONE-D-23-16077

**Title:** Early Neonatal Mortality and Determinants in sub-Saharan Africa: Findings from Recent Demographic and Health Survey data

**Journal:** PLOS ONE

**Subject:** Submission of revised manuscript

We thank the editors facilitating this manuscript for possible publication and the reviewers for their time spent reviewing the manuscript and their insightful comments and suggestions that helped improve the quality of this manuscript. The comments are encouraging, and the reviewer appears to share our judgment that this study and its findings are significant from a scientific standpoint. Please see our thorough answer to the comments below. Please refer to the revised manuscript file that has been supplied separately in the attachment.

Yours sincerely,

Tadesse Tarik Tamir, corresponding author (on behalf of all authors)

University of Gondar, Gondar, Ethiopia

**Response to editors and reviewers’ and comments**

PLOS ONE does not copyedit accepted manuscripts, so the language in submitted articles must be clear, correct, and unambiguous. Any typographical or grammatical errors should be corrected at revision, so please note any specific errors here.

Response: Thank you. The authors considered proofreading the manuscript and corrected any typographical or grammatical errors.

**Reviewer #1:** The sentence numbers should be continuous from title to references rather than breaking them down per section because it can create confusion.

Response: Dear reviewer, the revised manuscript is provided with the continuous line numbers. Thank you.

Title ; Capitalise every word that is more than three words in the title

Response: Thank you. We fully addressed the concern.

Referencing…. cite ALL the reference numbers in square brackets.

Response: Dear reviewer, taking your comments into account we cited the reference numbers in square brackets. Kindly find the point in our revised manuscript.

Line 15 of Statistical Analysis … there is need to work on the verb
Once extracted from the source, the data for each country has been assembled, m

Response: Dear reviewer, thank you for the constructive comments. The sentence is now amended for clarity to “Once extracted from the source, the data from each country has been appended together to create one single file dataset using Stata 14.”

• Use of Kids Records is not the most appropriate word to use unless it was officially
called that as in the statement .. The word Kids is used when dealing with animals.

Response: Dear reviewer, thank you for your insightful comment. We made modifications to the statement in revised manuscript. Thanks to your constructive comment, it is now changed to “This study used the children’s record dataset to determine the outcome variable.”

RESULTS
Line 28… it should be Results.

Response: Thank you for the comments. We addressed it accordingly. Kindly find it in our currently revised manuscript.

Revise of the categories of ANC visits in table 2 in because they seem to mean the same thing.
ANC visits
>4 visits
≥4 visits

Response: Dear reviewer, thank you for your correction. The point is now addressed accordingly.
In Table 2, Please write the fourth category of Birth order in words to maintain consistency in

Response: Dear reviewer, the comment is invaluable and it is now addressed accordingly. Please find it in the revised manuscript.

Regarding the Mode of delivery variable in Table 2 and Table 4:…. the category (CS) should be written in full.

Response: Thank you, the comment is addressed accordingly. Kindly find the point in our revised manuscript.

In the discussion section…. improve on the discussion of the variable under Line 17 to 21
Response: Dear reviewer, thank you for your scientifically invaluable feedback. We made improvements to the section as per your comment. Kindly find the point in our revised manuscript.

Limitations… since the data from the different countries was collected in different years, couldn’t this have an effect on the results. if so please include it in the limitations

Response: Dear reviewer, thank you for such an insightful concern. The concern is now addressed by including it in the limitation section of the revised manuscript. Please see the limitation section of our revised manuscript.

**Reviewer #2:** The authors have addressed all of the comments. However, I still think that manuscript can benefit from additional language editing.

Response: Thank you. The authors considered proofreading the manuscript and corrected any typographical or grammatical errors.

We thank you for your insightful comments, suggestions, and guidance, which have significantly improved the study.
