# Supplementary material for: Early neonatal mortality and determinants in sub-Saharan Africa: Findings from recent demographic and health survey data
Source: PLoS One. 2024 Jun 7;19(6):e0304065. doi: 10.1371/journal.pone.0304065 (PMC11161111; doi:10.1371/journal.pone.0304065)
Supplement: Attachment — Submitted filename: Rebutal letter.docx [file pone.0304065.s004.docx]

**Response to comments**

**Manuscript ID:** PONE-D-23-16077

**Title:** Early neonatal mortality and determinants in sub-Saharan Africa: findings from recent Demographic and Health Survey data

**Journal:** PLOS ONE

**Subject:** Submission of revised manuscript

We thank the editors facilitating this manuscript for possible publication and the reviewers for their time spent reviewing the manuscript and their insightful comments and suggestions that helped improve the quality of this manuscript. The comments are encouraging, and the reviewer appears to share our judgment that this study and its findings are significant from a scientific standpoint. Please see our thorough answer to the comments below. Please refer to the revised manuscript file that has been supplied separately in the attachment.

Yours sincerely,

Tadesse Tarik Tamir, corresponding author (on behalf of all authors)

University of Gondar, Gondar, Ethiopia

**Response to reviewer comments**

The spacing between in text references and words in sentences line 249_251

**Response**: Dear reviewer, we addressed the comment accordingly.

References in the Reference list should follow PLos One recommendations with Authors name being first before author initials.

**Response**: Dear reviewer, we provided the revised manuscript with the list of references as per PLOS ONE recommendation. Thank you!

Bold the title for the figure 1.

**Response**: Dear reviewer, Thank you. We addressed the concern accordingly.
